# Inhibition of PDE10A in a New Rat Model of Severe Dopamine Depletion Suggests New Approach to Non-Dopamine Parkinson’s Disease Therapy

**DOI:** 10.3390/biom13010009

**Published:** 2022-12-21

**Authors:** Ilya Sukhanov, Artem Dorotenko, Zoia Fesenko, Artem Savchenko, Evgeniya V. Efimova, Mikael S. Mor, Irina V. Belozertseva, Tatyana D. Sotnikova, Raul R. Gainetdinov

**Affiliations:** 1 Valdman Institute of Pharmacology, Pavlov First St. Petersburg State Medical University, 197022 St. Petersburg, Russia; 2 Institute of Translational Biomedicine, St. Petersburg State University, 199034 St. Petersburg, Russia; 3 St. Petersburg University Hospital, St. Petersburg State University, 199034 St. Petersburg, Russia

**Keywords:** PDE10A inhibitors, locomotor activity, hypodopaminergia, Parkinson’s disease, dopamine transporter knockout rat

## Abstract

Parkinson’s disease is the second most common neurodegenerative pathology. Due to the limitations of existing therapeutic approaches, novel anti-parkinsonian medicines with non-dopamine mechanisms of action are clearly needed. One of the promising pharmacological targets for anti-Parkinson drug development is phosphodiesterase (PDE) 10A. The stimulating motor effects of PDE10A inhibition were detected only under the conditions of partial dopamine depletion. The results raise the question of whether PDE10A inhibitors are able to restore locomotor activity when dopamine levels are very low. To address this issue, we (1) developed and validated the rat model of acute severe dopamine deficiency and (2) tested the action of PDE10A inhibitor MP-10 in this model. All experiments were performed in dopamine transporter knockout (DAT-KO) rats. A tyrosine hydroxylase inhibitor, α-Methyl-DL-tyrosine (αMPT), was used as an agent to cause extreme dopamine deficiency. In vivo tests included estimation of locomotor activity and catalepsy levels in the bar test. Additionally, we evaluated the tissue content of dopamine in brain samples by HPLC analysis. The acute administration of αMPT to DAT-KO rats caused severe depletion of dopamine, immobility, and catalepsy (Dopamine-Deficient DAT-KO (DDD) rats). As expected, treatment with the L-DOPA and carbidopa combination restored the motor functions of DDD rats. Strikingly, administration of MP-10 also fully reversed immobility and catalepsy in DDD rats. According to neurochemical studies, the action of MP-10, in contrast to L-DOPA + carbidopa, seems to be dopamine-independent. These observations indicate that targeting PDE10A may represent a new promising approach in the development of non-dopamine therapies for Parkinson’s disease.

## 1. Introduction

Parkinson’s disease (PD) is the second most common neurodegenerative pathology after Alzheimer’s disease [1]. The number of worldwide PD cases is estimated to increase from about 4.1 million in 2005 to nearly 8.7 million by 2030 [2]. The development of the disease is clearly age-dependent; its prevalence increases with age and peaks between ages 85 and 89 [2,3].

Despite major efforts from scientists, the exact cause of sporadic PD remains enigmatic. Nonetheless, we know that PD development is associated with progressive irreversible loss of dopamine (DA) neurons in the substantia nigra [4]. The common symptoms of PD include motor disturbances (rigidity and akinesia), as well as gastrointestinal, autonomic problems, and cognitive deficits. Since the introduction of L-DOPA in clinics [5], this drug (in combination with carbidopa, the inhibitor of aromatic-L-amino-acid decarboxylase) remains the golden standard of PD treatment. However, chronic replacement therapy with L-DOPA is associated with many adverse reactions that develop with time. Such adverse reactions include, first of all, motor fluctuations and dyskinesias, developing in 40–75% of patients [6,7]. Hallucinations, impulse-control disorders, and DA dysregulation syndrome are also adverse reactions to L-DOPA treatment [6,7]. Other anti-parkinsonian drugs include mostly agents also affecting DA neurotransmission: DA receptor agonists (rotigotine and apomorphine), inhibitors of monoamine oxidase B (selegiline and rasagiline), and catechol-o-methyltranferase (tolcapone and entacapone) [8]. However, these drugs are mainly used as adjuvants to L-DOPA therapy [8]. In sum, due to these limitations of existing therapeutic approaches, novel anti-parkinsonian medicines with non-DA mechanisms of action are clearly needed.

A promising pharmacological target for anti-parkinsonian drug development is phosphodiesterase (PDE) 10A [9,10]. PDE10A belongs to the large family of PDEs, enzymes degrading cAMP and cGMP and therefore playing a pivotal role in the regulation of intracellular signaling [11]. The important feature of PDEs is the unique tissue-specific pattern of their expression, which provides a great opportunity for pharmacological manipulations [12]. PDE10A is expressed nearly exclusively in the striatal medium spiny neurons (MSNs) [13,14,15,16,17,18]. Two populations of MSNs give rise to the direct and the indirect pathways of movement control [13,19,20,21,22]. Given that the regulation of cAMP synthesis is an important aspect of DA receptor signaling [23], and due to the specific localization in striatal MSNs, PDE10A inhibitors are supposed to be able to influence DA-ergic transmission at the intracellular level.

It is well-known that the activation of D1-like DA receptor (D1R) results in increased cAMP levels, whereas D2-like DA receptor (D2R) activation causes a decreased intracellular concentration of this secondary messenger [23]. Because PDE10A is present in both D1- and D2R-expressing populations of MSNs, its inhibitors may functionally imitate the actions of both D1R agonists and D2R antagonists. The inhibition of PDE10A in intact animals results in motor suppression and other effects typically observed following D2R antagonist administration [24,25,26,27,28]. The stimulating motor effects of PDE10A inhibition in D1R-expressing MSNs can be detected only under the conditions of partial DA depletion caused by vesicular monoamine transporter inhibition, as well as a D1R or D2R blockade [29,30]. The promising results demonstrated in these studies raise the question of whether PDE10A inhibitors are able to restore locomotor activity independently from DA. An in vivo model for the lack of striatal DA was developed previously in DA transporter knockout (DAT-KO) mice, in which striatal DA levels, due to the absence of DA reuptake, become fully dependent on the ongoing synthesis of DA. Administration of α-Methyl-DL-tyrosine (αMPT), a tyrosine hydroxylase inhibitor, to DAT-KO mice causes the elimination of striatal DA, immobility, and catalepsy (DA-deficient DAT-KO mice; DDD mice) [31]. By comparison, in wild-type mice treated with αMPT, only 50% depletion of striatal tissue DA levels and minimal behavioral manifestations were observed [31]. This model of acute severe DA deficiency is routinely used to screen for potential anti-parkinsonian drugs [31,32]. To address this issue, we (1) developed and validated an analogous rat model of acute severe DA deficiency (DA-deficient DAT-KO (DDD) rats) and (2) tested the action of PDE10A inhibitor MP-10 in this model.

## 2. Materials and Methods

### 2.1. Subjects

Experiments were performed in the animals that originated from the previously described rat strain with a loss-of-function mutation of the DAT gene [33,34]. Drug- and test-naïve female DAT-KO rats from the local colony of the Valdman Institute of Pharmacology were housed under a 12 h/12 h light/dark cycle (lights on at 08:00 h) at 21 ± 2 °C and 50 ± 20% humidity. Rats (>150 g at the beginning of the experiments) were housed in groups of same-sex siblings (3–5 per TIV cage (Tecniplast, Italy)). During the experiments, animals had free access to filtered (“AQUAPHOR”, Saint Petersburg, Russia) tap water and standard laboratory rat chow (receipt ПK 120-1, KKZ “Laboratorkorm”, Moscow, Russia). The cages, corn cob bedding (“KKZ ‘Zolotoy pochatok’” LLC, Voronezh, Russia), and water bottles were changed once a week.

All tests were performed during the light period of the light/dark cycle. Experimental protocols were approved by the Local Animal Care and Use committee (First Pavlov State Saint Petersburg Medical University, #100_ИΦ1_012017/3_900 and #100_ИΦ1_012019/21_300).

### 2.2. Compounds

In the present study, αMPT (α-Methyl-DL-tyrosine methyl ester hydrochloride; Sigma-Aldrich; CAS#: 7361-31-1), a tyrosine hydroxylase inhibitor, was used as an agent to cause DA deficiency in DAT-KO rats. The compound was dissolved in distilled water and administered intraperitoneally (i/p) in a 250 mg/kg dose (dosing volume—2 mL/kg).

To evaluate the predictive validity of the DDD protocol, we used the combination of two compounds: L-DOPA (3-(3,4-dihydroxyphenyl)-L-alanine; Fluka analytical; CAS#: 59-92-7) and carbidopa ((S)-3-(3,4-Dihydroxyphenyl)-2-hydrazino-2-methylpropanoic acid; Sigma-Aldrich; CAS#: 38821-49-7). Previously published literature was considered to choose the dose range, starting with 10 mg/kg for both agents [35]. L-DOPA was dissolved in acidified distilled water (pH = 4). A concentration of 0.1% *w*/*v* Tween 80 (“KKZ “Arcona SPb””, Saint Petersburg, Russia) in acidified distilled water (pH = 2) was used as a vehicle to prepare the carbidopa solution. For both preparations, pH was adjusted with hydrochloric acid. Fresh solutions were prepared before tests and administered i/p in the volume of 20 ml/kg and 5 ml/kg for L-DOPA and carbidopa, respectively.

MP-10 (2-[[4-(1-methyl-4-pyridin-4-ylpyrazol-3-yl)phenoxy]methyl]quinoline hydrochloride; Sun-Shine Chem; CAS#: 898562-94-2) was taken as a PDE10A inhibitor. MP-10 was dissolved in 10% Tween 80 (“KKZ “Arcona SPb””, Saint Petersburg, Russia). Fresh solutions were prepared before tests and administered i/p in the volume of 1 mL/kg. The tested doses of MP-10 (1, 3, 5 mg/kg) were chosen according to previously published data [29,36].

### 2.3. Design of Experiments

The motor effects of L-DOPA + carbidopa (0 + 0; 10 + 10; 20 + 10; 40 + 10 mg/kg) and PDE10A inhibitor MP-10 (0; 1; 3; 5 mg/kg) were estimated on independent experimental groups. Simple randomization was used for the unbiased assignment of subjects to treatment groups during in vivo tests. Blinded experimenters performed dosing procedures in the experiment with L-DOPA + carbidopa. MP-10 administration was conducted without blinding. The bioanalytical part of the experiments was performed by blinded experimenters. The timelines of procedures in detail for both experiments are shown in Table 1.

After completing the in vivo experiments, the animals were sacrificed by cervical dislocation by trained staff, and brain samples were collected for neurochemical analysis.

### 2.4. In Vivo Tests

#### 2.4.1. Locomotor Activity

To assess locomotion, two sets of five identical Plexiglas boxes (25 cm × 35.5 cm × 34 cm) with transparent walls were used. Each set of boxes was enclosed within sound-attenuating ventilated cubicles; the light intensity inside the apparatus was 30–40 lx. Each locomotor activity box was equipped with 11 pairs of photocell-based infrared sensors. Three pairs of infrared sensor units located 5 cm above the bottom of the box and eight pairs of units located 14 cm above the bottom of the box were used to record horizontal and vertical movements, respectively. Boxes’ inputs were connected to an operating PC equipped with Med-PC software through the MED interface (MED Associates, East Fairfield, VT, USA). The number of the sequential beam breaks (ambulation) was recorded for each animal as the measure of horizontal locomotor activity.

#### 2.4.2. Bar Test

To assess catalepsy levels in rats, the “bar test” was performed. The procedure has been described in detail before [37]. The forepaws of rats were gently placed on the horizontal bar (1.0 cm diameter smooth, wooden rod attached to plastic supports) situated 10 cm above the surface, and descent latency (time in seconds from when the forepaws were placed on the rod until the rat touched the floor by its forepaw or its hind paws left the floor) was recorded. We used a 180 s cut-off time, meaning the trial was terminated when the animal did not make an active paw movement within that time.

### 2.5. Ex Vivo Bioanalytical Procedures

The striatum and the frontal cortex (FC) of animals were dissected on ice, frozen in liquid nitrogen, and stored at −80 °C. For analysis, the samples were homogenized in 0.1 M HClO_4_ (20 μL per mg of tissue) containing 100 ng/mL 3,4-dihydroxybenzylamine (DHBA) as an internal standard. Homogenates were centrifuged for 10 min at 10,000× g (at +4 °C). Supernatants were filtered through centrifuge filter units [polyvinylidene fluoride (PVDF) membrane; pore size 0.22 μm, Millipore, Burlington, MA, USA] and then analyzed for levels of DA, using HPLC with electrochemical detection (Eicom, HTEC-500, Japan) with a carbon electrode WE-3G (Eicom, Tokyo, Japan), using +650 mV applied potential. The separation was carried on a reverse-phase column CA-50DS (150 × 2.1 mm, Eicom, Japan) at 200 μL/min flow rate. The mobile phase contained 100 mM sodium-phosphate buffer, 0.17 mM EDTA, 1.8 mM octanesulfonic acid sodium salt, and 19.5% (vol/vol) methanol, pH 4.31. The volume of injection was 10 μL. All peaks obtained were normalized to internal standard 3,4-dihydroxybenzylamine, and final values were expressed as nanograms per milligram of wet tissue weight.

### 2.6. Statistical Analysis and Plots

Data manipulations and visualization were performed in the R software environment (R: The R Project for Statistical Computing).

Locomotor activity and catalepsy scores were analyzed by mixed-model analysis of variance (mixed ANOVA) with the Satterthwaite method for degrees of freedom, using the “lmerTest” package [38] (version 3.1.3). All dependent variables were rank-modified. Follow-up tests of significant main effects or interactions were conducted using the “emmeans” package (version 1.7.4.1). The Dunnett’s method for adjusting *p* values and the Kenward–Roger method for degrees of freedom were applied in multiple comparisons.

Additionally, survival analysis was applied to analyze the bar test’s data (see the Supplementary Material).

Non-parametric ANOVA (the Kruskal–Wallis (KW) test) was used to compare tissue DA concentrations. The Dunn’s test (“dunn.test” package, version 1.3.5) was performed for pairwise comparisons whenever significant results were indicated by the ANOVA. Alpha was set at 0.05.

## 3. Results

### 3.1. Treatment with L-DOPA + Carbidopa Restores Motor Functions and Brain DA Levels in DDD Rats

#### 3.1.1. Motor Functions

**Figure 1:**
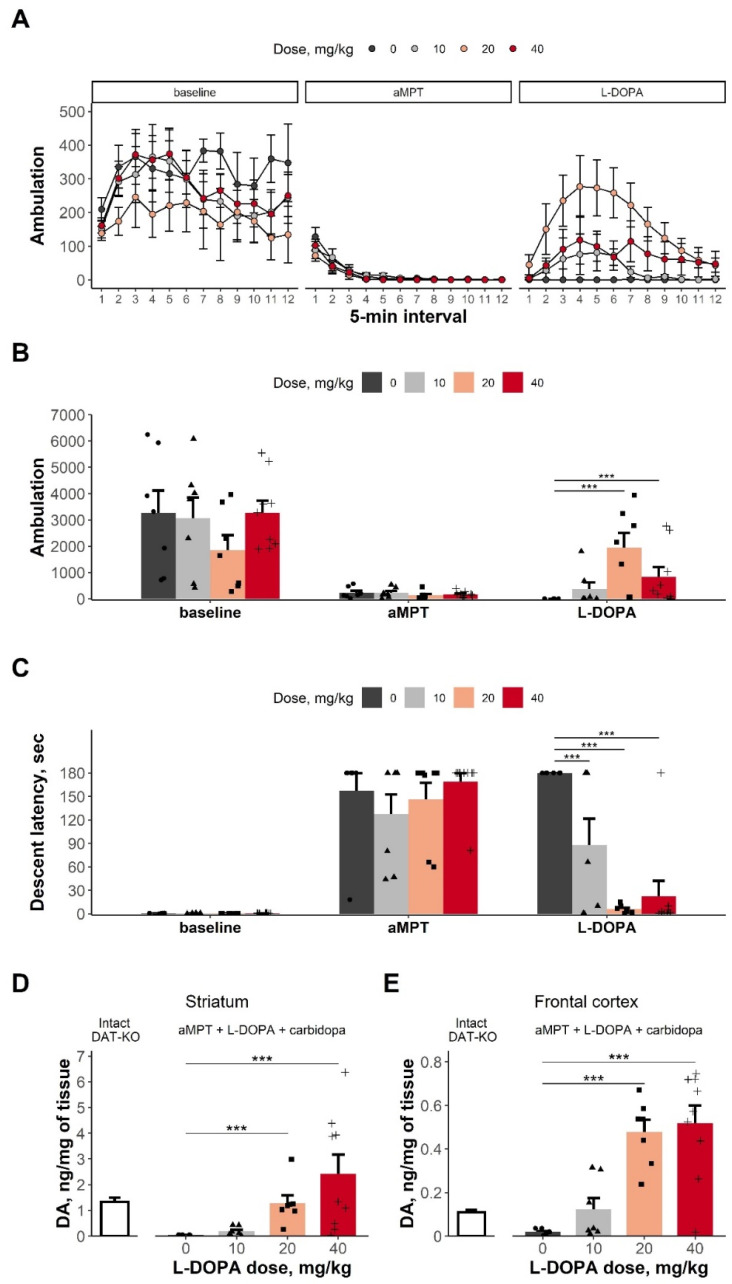
DA depletion, akinesia, and catalepsy in DDD rats. Treatment with L-DOPA + Carbidopa restores motor functions and brain DA levels in DDD rats. (**A**) Horizontal locomotor activity (ambulation) during different phases of the experiment (5-min intervals). (**B**) Horizontal locomotor activity (ambulation) during different phases of the experiment (total value). (**C**) Descent latency time before (the bar test I) and after (the bar test II) αMPT administration and 0.5 h after L-DOPA + carbidopa treatment (the bar test III). Postmortem levels of DA in striatum (**D**) and FC (**E**) of intact DAT-KO rats (baseline) and DDD rats after L-DOPA + carbidopa treatment. A sample from each animal was analyzed as a data point. Data are presented as mean + SEM. Individual meanings are presented as dot, triangle square and plus sign for corresponding treatment groups. ***—*p* < 0.001 compared with the vehicle control treatment, as analyzed using the Dunn’s *post hoc* test.

In concordance with the above-mentioned results, αMPT administration was accompanied by full immobility (Figure 1A,B). The ANOVA failed to detect a significant effect of ‘dose’ (F(3,26) = 2.024, *p* = 0.135); however, significant effects of ‘phase’ (baseline, αMPT or L-DOPA treatment) (F(2,52) = 60.073, *p* < 0.001) and of the interaction of these factors (F(6,52) = 6.725, *p* < 0.001) were found. The treatment with L-DOPA + carbidopa (20 + 10 and 40 + 10 mg/kg) restored animal locomotor activity (Dunnett’s test, Figure 1B).

Following αMPT treatment, prominent catalepsy in rats developed. This was reflected by increased descent latency in the bar test in any experimental group (Figure 1C). Subsequent L-DOPA treatment resulted in a significant decrease of descent latency at any tested dose (the ANOVA: the factor ‘dose’—F(3,78) = 6.561, *p* < 0.001; the factor ‘phase’—F(2,78) = 152.747, *p* < 0.001; the interaction—F(6,78) = 6.465, *p* < 0.001; Figure 1C).

#### 3.1.2. DA Levels in the Striatum and the FC

As expected, the treatment with 20 and 40 mg/kg of L-DOPA (+10 mg/kg carbidopa) was accompanied by a significant increase in DA tissue content, both in the striatum and in the FC (the KW test: the striatum—H = 17.349, df = 3, *p* < 0.001, Figure 1D; the FC—H = 17.907, df = 3, *p* < 0.001, Figure 1E).

### 3.2. Treatment with MP-10 Restores Motor Functions in DDD Rats without Affecting Brain DA Content

#### 3.2.1. Motor Functions

**Figure 2:**
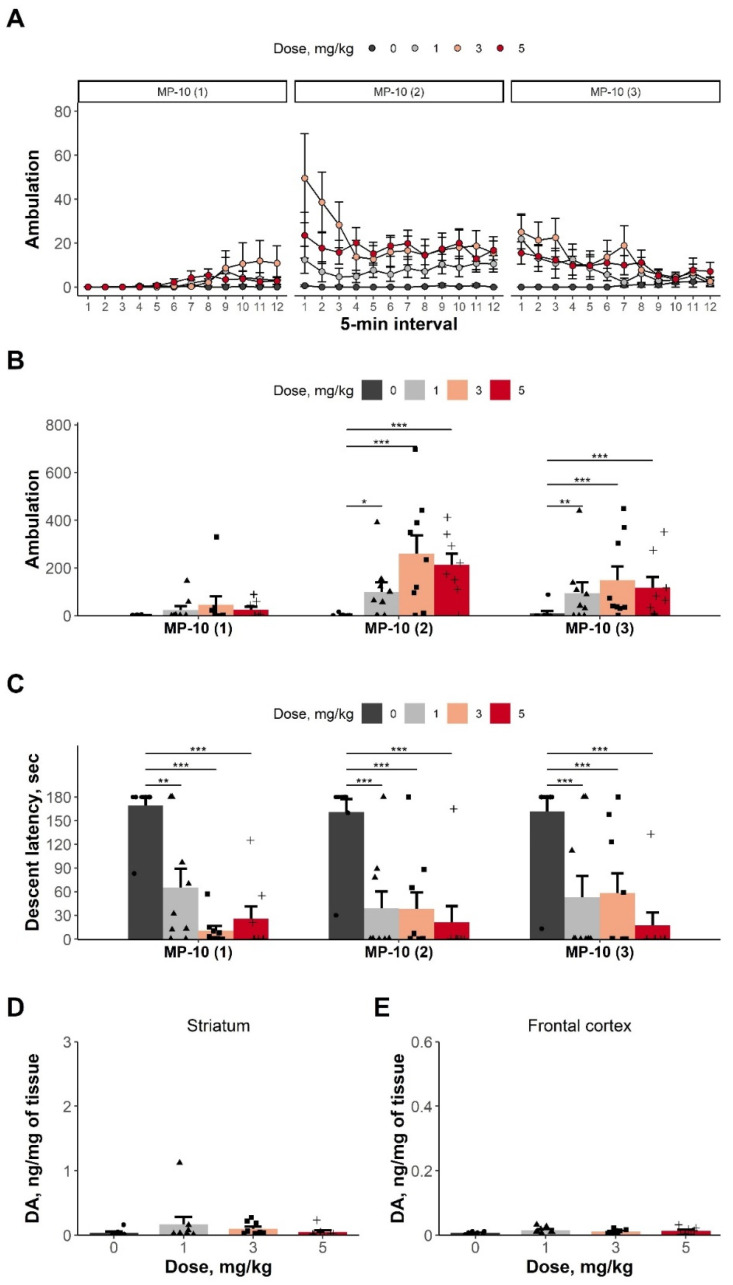
Treatment with MP-10 restores motor functions in DDD rats without affecting brain DA content. (**A**) Horizontal locomotor activity (ambulation) after MP-10 treatment during different phases of the experiment (5-min intervals). (**B**) Horizontal locomotor activity (ambulation) after MP-10 treatment during different phases of the experiment (total value). (**C**) Descent latency time of DDD rats 1.5 h, 2.5 h, and 3.5 h after MP-10 treatment (the bar tests III, IV, and V, respectively). Postmortem levels of DA in striatum (**D**) and FC (**E**) of DDD rats, following MP-10 treatment. A sample from each animal was analyzed as a data point. One FC sample was excluded due to error during the sampling procedure. Data are presented as mean + SEM. Individual meanings are presented as dot, triangle square and plus sign for corresponding treatment groups. *—*p* < 0.05, **—*p* < 0.01, ***—*p* < 0.001 compared with the vehicle control treatment, as analyzed using the Dunn’s *post hoc* test.

The effects of αMPT in new cohort of DAT-KO rats developed as described in Section 3.1.1 (see the Supplementary Materials for data). The MP-10 administration reversed akinesia; the ANOVA revealed significant effects of factor ‘dose’ (F(3,31) = 8.610, *p* < 0.001), ‘phase’ (F(4,124) = 85.719, *p* < 0.001), and their interaction (F(12,124) = 2.965, *p* < 0.01). A post hoc analysis detected that treatment with any MP-10 dose significantly increased horizontal activity of DDD rats during the second and third phases, compared to the vehicle group (the Dunnett’s test, Figure 2B).

Similarly to L-DOPA, the MP-10 administration in any tested dose was associated with a significant decrease in descent latency in the bar test (the ANOVA: factor ‘dose’—F(3,31) = 13.573, *p* < 0.001; factor ‘phase’—F(4,124) = 39.425, *p* < 0.001; the factors’ interaction—F(12,124) = 7.162, *p* < 0.001; Figure 2C).

#### 3.2.2. DA Tissue Content in MP-10 Treated DDD Rats

The MP-10 administration did not influence tissue DA concentration in the striatum or the FC (the KW test: the striatum—H = 3.584, df = 3, *p* = 0.31, Figure 2D; the FC—H = 4.564, df = 3, *p* = 0.21, Figure 2E).

## 4. Discussion

Our results demonstrated that the administration of αMPT caused striking immobility and catalepsy in rats lacking DAT. The treatment with either an L-DOPA and carbidopa combination or MP-10 restored the motor functions in DDD rats. According to neurochemical studies, the action of MP-10, in contrast to L-DOPA + carbidopa, seems to be DA-independent.

### 4.1. A Novel Model of Acute Severe DA Deficiency, DDD Rats

DA reuptake by the DAT transporter is an important element in the normal functioning of DA-ergic signaling, the significance of which is the regulation of the extracellular DA level. The DAT knockout rats and mice (DAT-KO lines) lack this reuptake mechanism, which leads to an accumulation of extracellular DA and, consequently, to hyperfunctioning of the brain DA system [33,39]. Animals of these lines are characterized by a striking phenotype—small size, hyperactivity, stereotypy, impairments of conditional associative learning, etc. [33,40,41,42].

Previously, using DAT-KO mice, the possibility of creating a state of temporary complete depletion of DA was demonstrated. Due to the lack of DA reuptake, striatal DA levels of these animals are fully dependent on ongoing synthesis. The pharmacological blockade of the DA synthesis enzyme tyrosine hydroxylase, using αMPT, leads to severe depletion of the DA pool, which is accompanied by striking changes in behavior; hyperactivity is replaced by akinesia and rigidity (freezing) that lasts for 16 hours [31,32,35]. A significant result of this work is the reproduction of the DDD mouse “zero DA” model in DAT-KO rats. We clearly demonstrated that pretreatment with αMPT resulted in akinesia and catalepsy development in the DAT-KO rats. Thus, DDD rats imitate akinesia and rigidity, some of key motor disturbances associated with PD in humans. As shown previously in DDD mice [31], DDD rats were also characterized by a severe deficiency of DA in the striatum and FC.

As in the case with DDD mice [31,32,35], DDD rats can become a new screening model for searching for new drugs for the treatment of PD. In our study, we used the combination of L-DOPA and carbidopa to validate our model for searching for compounds with anti-parkinsonian action. We demonstrated that the administration of the DA precursor combined with the inhibitor of aromatic-L-amino-acid decarboxylase resulted in the full restoration of DDD rats’ motor functions. It can be counterintuitive that the magnitude of locomotor activation after L-DOPA treatment with the highest used dose (40 mg/kg) seemed to be lower compared to the action of L-DOPA at a dose of 20 mg/kg. However, the most probable reason for this phenomenon may be the pronounced motor stereotypies in the rats treated with the highest dose of L-DOPA. These observed stereotypies can decrease the horizontal activity of the rats. The evaluation of the DA content in the striatum and the FC revealed that the restoration was accompanied by the elevation of brain DA. However, the opinion that L-DOPA acts only via increases in striatal DA levels seems to be an oversimplification of a very complex set of neurochemicals processes (for review see [43,44]). For example, L-DOPA administration is accompanied by an increase in 3-methoxytyramine levels. This DA metabolite is able to affect movement via interaction with Trace Amine-Associated Receptor 1 (TAAR1) [45,46].

The need for the emergence of another model that reproduces the pathological processes of PD does not require explanation. As described in the introduction, today there is an urgent need for an improvement in the existing therapy for PD. At the same time, the directions of research on non-DA-ergic treatment targets are becoming increasingly important [47,48,49,50]. The DDD rats presented in this study have certain advantages over corresponding mouse models of severe DA depletion. Rats have a larger brain size for surgeries and neuronal recordings, and they have better translational value, in many aspects being physiologically closer to humans [51].

### 4.2. DA-Independent Restoring of DDD Rats’ Motor Functions by PDE10A Inhibition

PDE10A is the important regulator of the adenylyl cyclase pathway in MSNs. Because this enzyme is present in both D1- and D2R expressing MSNs, functionally the inhibitors of PDE10A can mimic the action of D2 receptor antagonists and D1 receptor agonists. We found that the administration of PDE10A inhibitor MP-10 dose-dependently restored locomotor activity and reversed catalepsy in DDD rats lacking DA. The results of the present study provide strong support to previously shown evidence that the D1R agonist-like action of PDE10A inhibitors can be demonstrated under conditions of partial DA deficiency [29,30]. The performed tissue content analysis revealed that the locomotion-restoring action of MP-10 seems not to be mediated by an increase in brain DA levels. We detected only trace DA content in the striatum and FC of the rats treated with the PDE10A inhibitor.

It should be noted, however, that treatment with even the highest tested dose of MP-10 did not result in as prominent locomotor hyperactivity as what was shown in DDD rats injected with L-DOPA + carbidopa. At the same time, the levels of locomotor activity of DDD rats treated with MP-10 was more or less the same as the locomotor activity of intact WT female rats (DAT-KO treated with MP-10 3 mg/kg (II) vs. first hour of WT female rat: 259.7 ± 76.8 vs. 328.0 ± 25.0). We can speculate that the L-DOPA-induced hyperactivity is determined by the agonistic action of DA on both the D1- and the D2R, whereas stimulation action of MP-10 seems to be caused only by its functional D1-agonistic action.

The findings of our study raise two extremely intriguing questions. First, as mentioned above, PDE10A inhibition increases the activity of both the direct (functional D1R agonism) and the indirect pathways (functional D2R antagonism). Thus, it seems intriguing as to why PDE10A inhibitors’ action on locomotor activity is opposite in usual and DA-depleted states.

One must acknowledge the complex interactions between the direct and indirect pathways. Some theories implicate the coordinated action of both pathways in the process of movement initiation and action selection [52,53,54,55,56,57,58]. We assume that under the condition of unaffected DA neurotransmission, it inhibits predominantly the indirect pathway, perhaps, because of the higher DA affinity to D2R than to D1R [59,60]. Therefore, PDE10A inhibition in D2R MSNs blocks DA inhibitory action on the indirect pathway that finally results in hypolocomotion. In the case of decreased DA transmission (DDD rats, tetrabenazine- or haloperidol-treated animals), cAMP levels in D2R MSNs’ increase, and it is possible that PDE10A inhibition cannot additionally stimulate the already increased tone of the indirect pathway and, hence, affects locomotion through this pathway. We can speculate that there is(are) some rate-limiting step(s) (e.g., protein kinase A activation) in cAMP signaling. The full activation of the indirect pathway seems to allow us to observe the stimulatory action of PDE10A inhibitors on the (D1R) direct pathway.

Second, which factors can activate adenylate cyclase and the production of cAMP (or cGMP) in D1R MSNs under conditions of severe DA depletion in DDD rats? We cannot fully exclude that a trace amount of DA is enough to induce cAMP accumulation, amplified by PDE10A inhibition. However, it is well-known that MSNs not only express DA receptors that can affect cAMP levels. For example, the MSNs of the direct pathway express adenosine A1, whereas the MSNs of the indirect pathway express both adenosine A1 and A2a receptors [61,62]. Both populations of the striatal MSNs express muscarinic (M1 and M4) and cannabinoid (CB1) receptors [63,64]. Cannabinoid CB1 and adenosine ones’ activation affect cAMP signaling pathways: the activation of adenosine A1, as well as CB1, receptors results in the inhibition of adenylate cyclase and reduction of cAMP levels, while the activation of adenosine A2a receptors causes elevation of cAMP levels [65]. Because of their pattern of expression, it seems unlikely that the adenosine A2a receptors that induced cAMP production can be responsible for the stimulating action of MP-10 in DDD rats. Moreover, the detailed GPCR receptor profile of MSNs has not been fully defined yet. For example, recent data indicate that TAAR1, TAAR6, and CRF1 are also expressed in the striatum [66,67,68,69]. Signaling of these receptors is associated with an increase in cAMP levels [70,71]. It should also be noted that some population of striatal MSNs express both D1 and D2 receptors [72] and how PDE10 inhibition can affect cAMP levels in these neurons and their contribution to the regulation of locomotion remain poorly understood. In sum, further studies aimed at the analysis of potential mechanisms of cAMP elevation in the D1R-expressing MSNs are strongly warranted.

### 4.3. Limitations

First, in the present project, we estimated the effects of the pharmacological agents in female DAT-KO rats. Despite the fact that we did not find studies demonstrating that the estrous cycle of rats can modulate the effects of the drugs, we cannot exclude that possibility. Second, following the principles of the 3R, we interrupted the measurement of locomotor activity by the bar test’s performance to reduce the number of used animals. Theoretically, our intervention can impact the results of locomotor activity measurements, although similar action did not affect the movement of vehicle-treated DDD rats.

## 5. Conclusions

In the present study, we developed and validated the rat model of acute severe DA deficiency, which can be used to test new medicines for the symptomatic treatment of PD. Using this model, we provided preclinical evidence that inhibition of the PDE10A enzyme may restore motor function in model animals independently of DA.

## Figures and Tables

**Table 1:** Timelines and procedure details of performed experiments.

Name of Experiment and Subjects	Phase	Procedures Order
MOTOR EFFECTS OF L-DOPA + CARBIDOPA IN THE DDD RATS DAT-KO rats (females, n = 30) Group 1 (n = 7)—0 + 0 mg/kg Group 2 (n = 7)—10 + 10 mg/kg Group 3 (n = 7)—20 + 10 mg/kg Group 4 (n = 9)—40 + 10 mg/kg	I. Baseline	(1) Locomotor activity test (60 min) (2) Bar test
	II. αMPT	(1) αMPT administration. Doses: 250 mg/kg, i/p (2) Locomotor activity test (60 min) (3) Bar test
	III. L-DOPA	(1) L-DOPA + carbidopa administration. Doses: 0 + 0, 10 + 10, 20 + 10, 40 + 10 mg/kg, i/p (2) Locomotor activity test (30 min) (3) Bar test (4) Locomotor activity test (30 min)
	Euthanasia, brain sampling for neurochemical analysis	
MOTOR EFFECTS OF PDE10A INHIBITOR IN THE DDD RATS DAT-KO rats (females, n = 35) Group 1 (n = 9)—0 mg/kg Group 2 (n = 9)—1 mg/kg Group 3 (n = 9)—3 mg/kg Group 4 (n = 8)—5 mg/kg	I. Baseline	(1) Locomotor activity test (60 min) (2) Bar test
	II. αMPT	(1) αMPT administration. Doses: 250 mg/kg, i/p (2) Locomotor activity test (60 min) (3) Bar test
	III. MP-10 (1)	(1) MP-10 administration. Doses: 0, 1, 3, 5 mg/kg, i/p (2) Locomotor activity test (60 min, postinjection time—30 min) (3) Bar test
	IV. MP-10 (2)	(1) Locomotor activity test (60 min) (2) Bar test
	V. MP-10 (3)	(1) Locomotor activity test (60 min) (2) Bar test
	Euthanasia, brain sampling for neurochemical analysis	

αMPT—α-Methyl-DL-tyrosine (tyrosine hydroxylase inhibitor); DDD—DA deficient DAT-KO rats.

## Data Availability

All of the data is presented in the article and Supplementary Materials. No additional data is reported.

## Supplementary Materials

The following supporting information can be downloaded at: https://www.mdpi.com/article/10.3390/biom13010009/s1, Figure S1: The effects of the pretreatment with L-DOPA + Carbidopa in DDD rats (10–40 mg/kg + 10 mg/kg, i.p.) on the distribution of the descent latency in bar test; Figure S2: The effects of the pretreatment with MP-10 in DDD rats (1–5 mg/kg, i.p.) on the distribution of the descent latency in bar test (n = 8–9 in each experimental group); Figure S3: Motor performance of rats treated with MP-10 during baseline and aMPT phases (n = 8–9 in each experimental group).

## Chemical Compounds Studied in this Article

α-methyl-p-tyrosine, L-DOPA, carbidopa, MP-10.
