# Supplementary material for: Inhibition of PDE10A in a New Rat Model of Severe Dopamine Depletion Suggests New Approach to Non-Dopamine Parkinson’s Disease Therapy
Source: Biomolecules. 2022 Dec 21;13(1):9. doi: 10.3390/biom13010009 (PMC9855999; doi:10.3390/biom13010009)
Supplement: Supplementary file 1 [file biomolecules-13-00009-s001.zip › biomolecules-2022082-supplementary.pdf]

Survival analysis of bar test's data

To additionally analyze the bar test's data we used fitting survival curves and following multiple comparisons using the log-rank test. Survival curves for each tested dose were compared within each phase separately. p values were adjusted with Bonferroni's correction.

L-DOPA

During the baseline phase there were no differences between survival curves in the bar test among the experimental groups (the log-rank test: χ^2=0.8, df=3, p=0.9). Following the aMPT treatment, prominent catalepsy in rats developed. This was reflected by increased probability to stay in the initial pose without differences between the groups (the log-rank test: χ^2=3.4, df=3, p=0.3) (Supplementary Figure S1). Subsequent L-DOPA treatment resulted in significant decrease of this measure (the log-rank test: χ^2=16.3, df=3, p=0.001). Pairwise comparisons revealed significant effects of L-DOPA + carbidopa treatment in doses 20 + 10 and 40 + 10 mg/kg compared to the vehicle group (p<0.001 and p<0.01, respectively; Supplementary Figure S1).

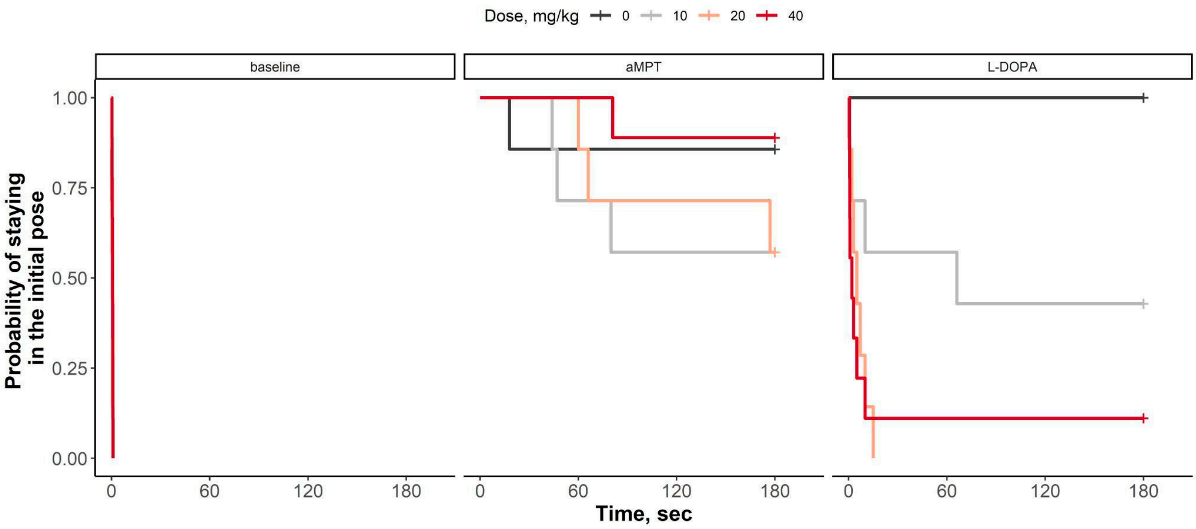

Supplementary Figure S1. The effects of the pretreatment with L-DOPA + Carbidopa in DDD rats (10-40 mg/kg + 10 mg/kg, i.p.) on the distribution of the descent latency in bar test (n = 7-9 in each experimental group). Data are represented as the Kaplan-Meier plot of the latency to stay in the initial pose.

MP-10

Similarly to L-DOPA, the MP-10 administration in any tested dose was associated with the significant decrease in probability of staying in the initial pose in the bar test (the log-rank test - phase “MP-10 (1)”: χ^2=23.9, df=3, p<0.001; 1 mg/kg vs vehicle p<0.05, 3 mg/kg vs vehicle p<0.001, 5 mg/kg vs vehicle p<0.01; phase “MP-10 (2)”: χ^2=16.0, df=3, p=0.01; 1 mg/kg vs vehicle p<0.05, 3 mg/kg vs vehicle p<0.001, 5 mg/kg vs vehicle p<0.001; phase “MP-10 (3)”: χ^2=19.4, df=3, p<0.001; 1 mg/kg vs vehicle p<0.05, 3 mg/kg vs vehicle p<0.01, 5 mg/kg vs vehicle p<0.001; Supplementary Figure S2).

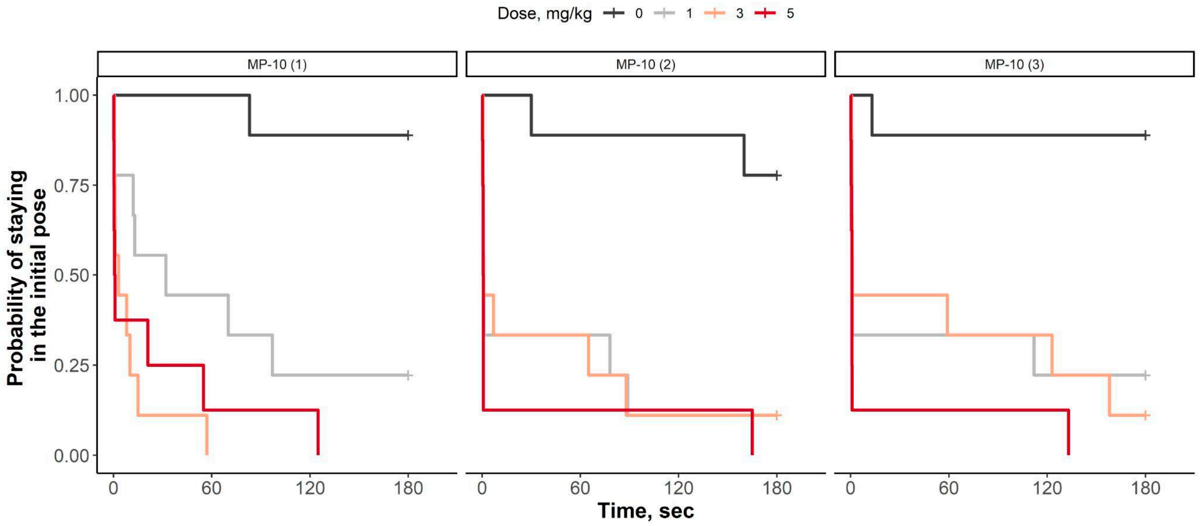

Supplementary Figure S2. The effects of the pretreatment with MP-10 in DDD rats (1-5 mg/kg, i.p.) on the distribution of the descent latency in bar test (n = 8-9 in each experimental group). Data are represented as the Kaplan-Meier plot of the latency to stay in the initial pose.

Baseline and aMPT phases MP-10

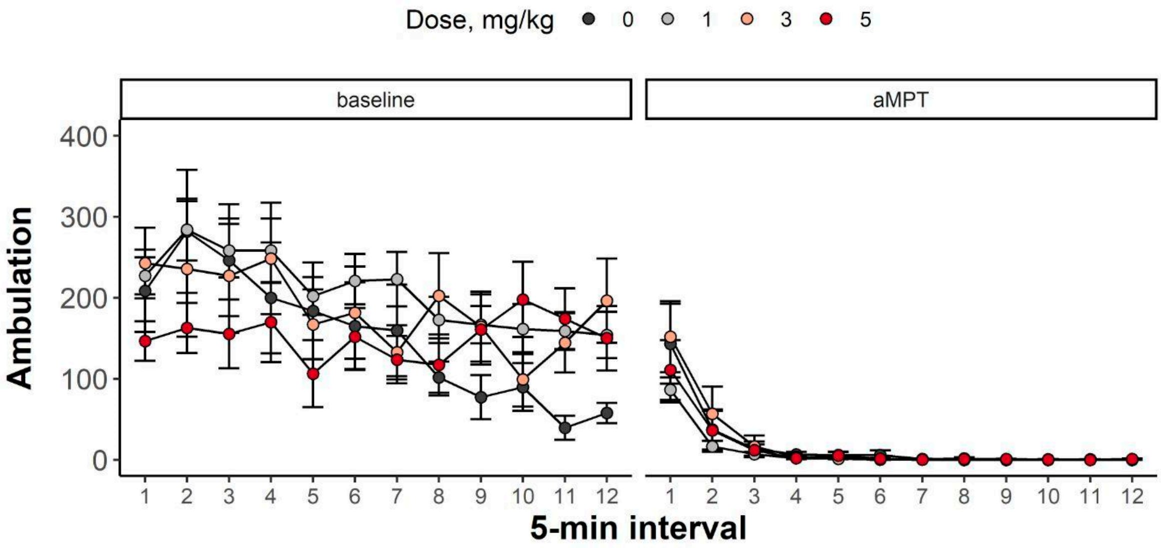

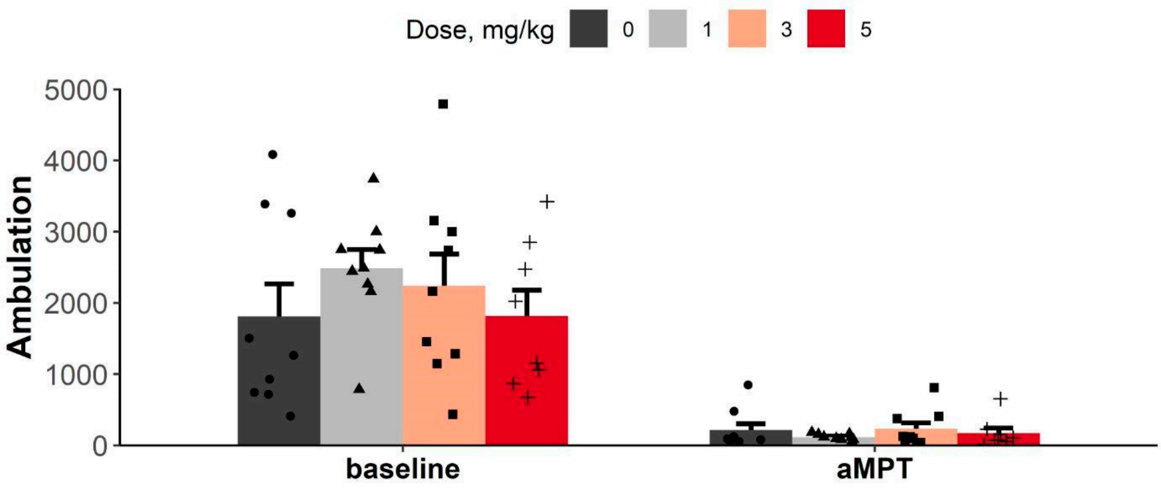

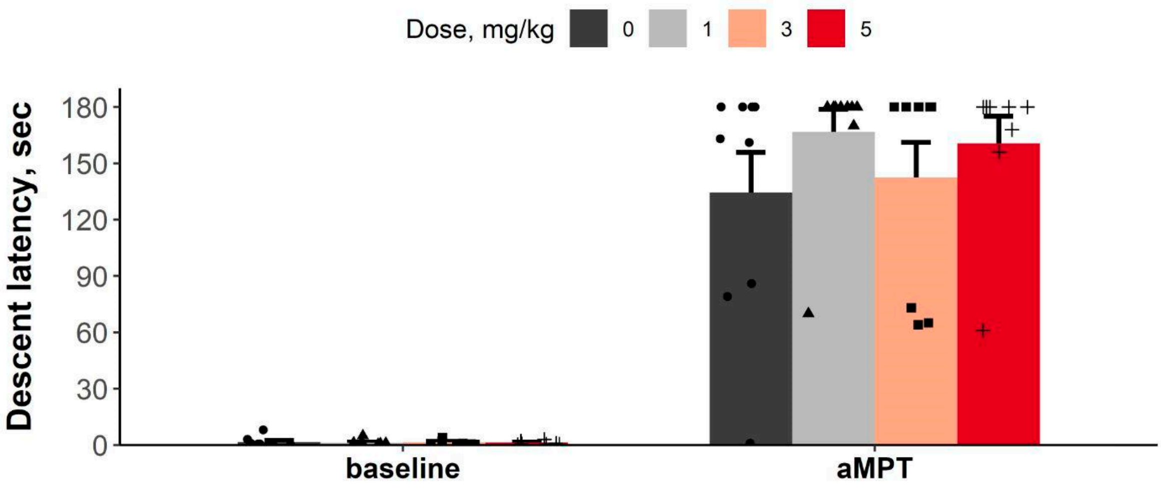

Supplementary Figure S3. Motor performance of rats treated with MP-10 during baseline and aMPT phases (n = 8-9 in each experimental group). Data are presented as mean + SEM. Additionally, individual values are plotted within each group.
